# Azole Resistance in *Aspergillus fumigatus* From Diverse Environments in Ohio, United States, Is Primarily Driven by TR_34_/L98H and TR_46_/Y121F/T289A Environmental Signatures

**DOI:** 10.1093/ofid/ofag150

**Published:** 2026-04-21

**Authors:** Raees A Paul, Sudharsan Sadhasivam, Timothy Frey, Sally A Miller, Pierce A Paul, Melanie L Lewis Ivey

**Affiliations:** Department of Plant Pathology, College of Food, Agricultural, and Environmental Sciences, The Ohio State University, Wooster, Ohio, USA; Department of Plant Pathology, College of Food, Agricultural, and Environmental Sciences, The Ohio State University, Wooster, Ohio, USA; Department of Plant Pathology, College of Food, Agricultural, and Environmental Sciences, The Ohio State University, Wooster, Ohio, USA; Department of Plant Pathology, College of Food, Agricultural, and Environmental Sciences, The Ohio State University, Wooster, Ohio, USA; Department of Plant Pathology, College of Food, Agricultural, and Environmental Sciences, The Ohio State University, Wooster, Ohio, USA; Department of Plant Pathology, College of Food, Agricultural, and Environmental Sciences, The Ohio State University, Wooster, Ohio, USA

**Keywords:** *Aspergillus fumigatus*, azole resistance, plant diseases, public health, surveillance

## Abstract

*Aspergillus fumigatus* is a leading global human fungal pathogen. Triazole antifungal drugs are used in clinical settings, while triazole demethylase inhibitor (DMI) fungicides are widely applied in the environment to combat plant fungal diseases. Environmental exposure to triazole fungicides may drive cross-resistance to clinical triazoles. To address limited data on environmental azole-resistant *A fumigatus* (ARAF) in the United States, we conducted surveillance across 75 sites in Ohio, including agricultural (57.3%), naturalized (25.3%), urban (16%), and commercial (1.3%) environments. Samples collected comprised air (n = 411), soil (n = 352), and compost (n = 42). Nested polymerase chain reaction of 397 airborne spore samples detected *A fumigatus* DNA in 62.7%, with 35.5% wild-type, 17.9% tandem repeat (TR), and 9.3% with mixed genotypes. TR genotype distribution did not differ by environment or DMI use. *Aspergillus fumigatus* was cultured from 18.1% of air samples, with 1.5% positive for ARAF. From soil and compost, *A fumigatus* was recovered from 41.4% of samples, of which 9.2% were positive for ARAF. Compost yielded higher *A fumigatus* (83.3%) and ARAF (21.4%) than soil (36.4% and 1.7%, respectively). ARAF prevalence was higher in urban (9.5%) than agricultural (2.8%) or naturalized (1.4%) environments. Generalized linear models suggested that compost and propiconazole exposure were significant predictors of ARAF occurrence. Among 72 ARAF isolates, 50% and 43% carried TR_34_/L98H and TR_46_/Y121F/T289A mutations, respectively, suggesting these mutations as key resistance signatures in Ohio environments. These findings identify compost and DMI exposure as a fertile milieu for ARAF development, highlight urban hotspots, and underscore the need for One Health approaches to resistance management.

## Teaser

This One Health study surveyed 75 Ohio environments and found azole-resistant *Aspergillus fumigatus* (ARAF) to be prevalent in compost and urban areas. TR_34_/L98H and TR_46_/Y121F/T289A mutations predominated, with compost and triazole fungicide exposure predicting ARAF occurrence, underscoring environmental reservoirs and cross-resistance risks to clinical azoles.

*Aspergillus fumigatus* is an environmental saprotrophic fungus that primarily contributes to the decomposition of plant matter and nutrient recycling. It is commonly found in soil, compost, and plant debris, releasing millions of airborne asexual spores [1]. When inhaled, spores can lead to a spectrum of lung infections, ranging from allergic and chronic lung conditions to life-threatening invasive aspergillosis (IA) [2]. Recent estimates indicate a global annual burden of 2.1 million cases of IA, with a crude mortality of 1.8 million [3].

The triazole compounds, belonging to the broader class of antifungal agents with azole chemistry, target a key fungal enzyme, lanosterol-14α-demethylase, in the ergosterol biosynthesis pathway, and are used to manage various clinical forms of *Aspergillus* disease [4]. Voriconazole is the first-line treatment for IA with isavuconazole as an alternative therapy [5]. For chronic pulmonary aspergillosis and allergic bronchopulmonary aspergillosis, itraconazole, a first-generation triazole, is one of the most prescribed agent for long-term oral antifungal therapy [6, 7]. Posaconazole is recommended as prophylaxis in high-risk patients with neutropenia, to prevent invasive mold infections, including IA [8]. The emergence of azole-resistant *A fumigatus* (ARAF) has serious implications for patients with IA with reported mortality rates ranging from 88% to 100% [9–11]. Given its significant global public health impact, *A fumigatus* was recently designated a “critical priority” pathogen by the World Health Organization [12]. In addition, ARAF poses a parallel threat to domestic animals, wildlife health, and food security, making it a significant One Health concern [13–15].

Azoles are broad-spectrum antifungal compounds that were introduced in the early 1970s for both clinical use and control of plant diseases [16]. The demethylase inhibitor (DMI) class of fungicides, characterized by either an imidazole or a triazole ring structure, are extensively used in agriculture, horticulture, turfgrass management, and wood preservation to control phytopathogenic and wood decaying fungi [17]. The broad-spectrum efficacy, cost-effectiveness, and versatility across a wide range of crop types makes azoles one of the leading fungicides used globally, with an estimated 48 000 metric tons of active ingredient applied to crops worldwide each year [18].

*Aspergillus fumigatus* can acquire resistance in vivo in context of a prolonged oral triazole therapy, especially in patients with chronic cavitary pulmonary aspergillosis [19]. In addition, resistance can emerge in broader One Health environments due to off-target exposure to triazole DMIs [15, 16]. The collateral exposure of *A fumigatus* to triazole DMIs outside the clinical settings is considered to drive the selection of ARAF, primarily associated with two canonical genetic signatures, TR_34_/L98H and TR_46_/Y121F/T289A, in the *cyp51A* gene encoding lanosterol-14α-demethylase [20]. Epidemiological and genomic evidence suggests that azole-naive patients acquire ARAF from hospital or community environments [21].

Although ARAF has emerged as a major clinical concern in Europe and Asia during the last decade, reports now suggest that ARAF is also a significant public health problem in the United States (US) [22]. In the US, surveillance studies on ARAF in environmental contexts have been limited to only a few studies [23–25]. Hurst et al [25] evaluated soil, fresh crop debris, and compost samples from four azole-sprayed peanut fields in a county in Georgia. While no ARAF was detected in soil or fresh crop debris, 19% of the isolates recovered from composted plant harvest debris were ARAF. Similarly, Kang et al [23] sampled agricultural sites across Georgia and Florida, and ARAF was recovered only from a composted pecan debris pile at a single site. A recent surveillance carried out at agriculture sites across five eastern and three western states showed widespread presence of ARAF with pan-azole-resistant isolates mostly from plant debris from vineyards, imported tulips, and hemp [24].

The patterns of azole use and exposure vary significantly across different environmental settings, making it crucial to assess the impact of DMI exposure on ARAF in diverse environments, both from a clinical perspective and in terms of food security. While cereal crops receive the highest total volumes of DMIs due to the large land acreage occupied by these crops, horticultural, ornamental crops, and turfgrass receive relatively intensive DMI applications [17].

To better understand ARAF prevalence and distribution in the US, we conducted a comprehensive cross-sectional surveillance of ARAF in air and soil samples from diverse environments across Ohio. We also compared the efficiency of phenotypic detection of ARAF and direct detection of *cyp51A* tandem repeat (TR) mutations with the long-term goal of identifying a high-throughput assay for the active surveillance of ARAF across a broad geographical range.

## MATERIALS AND METHODS

### Sampling Sites and Sample Collection

Between October 2022 and December 2024, air, soil, and compost samples were collected from 75 sites in Ohio. The sampling environments were classified into 4 broad categories: agricultural, urban, naturalized, and commercial (Supplementary Table 1). A total of 411 replicate air samples, 352 replicate soil samples, and 42 compost samples were collected from the study environments. Site-specific metadata were recorded using the AspTrack mobile application, developed on the noncode platform Clappia (https://dashboard.clappia.com/workplace/create). Collected metadata included GPS coordinates, environment type, details of DMI fungicide use during the current or preceding growing season, and the generic DMI compound applied. Air samples were actively collected into a sterile 1.5-mL Eppendorf tube using a custom-designed vacuum-assisted cyclonic sampler (Walt Mahaffee, Agricultural Research Service, US Department of Agriculture, personal communication). Soil and compost samples were collected in 58-mL Whirl-Pak bags (Whirl-Pack Filtration Group, WI, USA), and all samples were transported to a Biosafety Level 2 laboratory in ice chests, stored at 4°C, and processed within 2 weeks of collection. The air samples were suspended in 1 mL of sterile phosphate-buffered saline (PBS) (150 mM NaCl, 10 mM Na_3_PO4, pH 7.4) with 0.05% Tween-20 and vortexed for 30 seconds, and 200 µL was plated on Sabouraud dextrose agar (SDA) amended with streptomycin (100 µg/mL) and chloramphenicol (50 µg/mL). The plates were incubated at 43°C for 48 hours and noted for growth of *A fumigatus.*

### ARAF Detection Using Culture

A replica plate method was optimized to detect putative ARAF directly from soil and compost samples, as the high number of colonies isolated per sample made testing all isolates for ARAF using the minimum inhibitory concentration (MIC) method impractical. Five grams of soil or compost was suspended in 10 mL of PBS containing 0.05% Tween-20 in 50-mL tubes and vortexed. A 200-µL aliquot of the soil sample suspensions was plated onto SDA and SDA amended with 5 µg/mL of propiconazole (SDAP), then incubated at 43°C, and growth of *A fumigatus* was noted after 48 hours. Samples showing growth both on SDA and SDAP were considered ARAF positive. For air samples, which typically yielded fewer *A fumigatus* colonies, a modified approach was used. Air samples were suspended in 1 mL of sterile PBS with 0.05% Tween-20, vortexed for 15 seconds, and 200 µL plated on SDA amended with 50 µg/mL chloramphenicol and 100 µg/mL streptomycin. The individual colonies that grew on SDA were isolated and screened for ARAF by inoculating 25 µL of the spore suspension on SDAP.

### *cyp51A* TR Detection From Air and Soil Matrices

To detect *cyp51A* TR directly from the air and soil samples, DNA was extracted using the DNeasy Power Soil kit (Qiagen, Germantown, MD, USA) in accordance with manufacturer guidelines with modifications. The sample DNA extracts were probed for TR signature mutations in *cyp51A* promoter region using a nested polymerase chain reaction (PCR) assay as previously described with modifications [26, 27].

### Minimum Inhibitory Concentration Assays

The MICs of the DMI triazoles tebuconazole and propiconazole, as well as the clinical triazoles itraconazole, posaconazole, and voriconazole, were determined for putative ARAF (n = 72) and non-ARAF isolates (n = 20) using broth microdilution in accordance with the Clinical and Laboratory Standards Institute's (CLSI) M38 reference method [28]. The MICs of medical triazoles were interpreted based on the CLSI interpretive breakpoints [29].

### Detection of *cyp51A* Environmental Signature Mutations in ARAF Isolates

High molecular weight DNA was isolated from 72 ARAF isolates using MasterPure Complete DNA and RNA purification kit (LGC Biosearch Technologies, Petaluma, CA, USA) following the protocol described earlier with modifications [30]. Diluted DNA was screened for *cyp51A* mutations using the AsperGenius multiplex qPCR kit (PathoNostics B.V., Maastricht, The Netherlands). ARAF isolates that did not harbor any of the mutations captured by AsperGenius multiplex assay were sequenced using primers described by Kang et al [23]. The raw *cyp51A* sequence reads were mapped to *A fumigatus* Af293 *cyp51A* reference sequence NC 007197.1 and proofread in SeqMan Ultra software (DNAStar Inc, Madison, WI, USA). The FASTA sequences were analyzed on FunResDB database against the wild-type (WT) *A fumigatus* reference sequence AF338659 [31].

Additional details of each method are provided in the Supplementary Methods.

### Statistical Analysis

The association between the *cyp51A* TR genotype detected in air samples with the nested PCR and environmental variables, such as the type of environment and DMI use, was assessed using Pearson χ^2^ test or Fisher exact test as applicable. Binary logistic regression was employed to evaluate the odds of association between *cyp51A* TR genotype using nested PCR and variables including environment category, environmental subcategory, DMI exposure at the sampling location, a positive *A fumigatus* culture, and a positive ARAF culture from the air sample. The distribution of *A fumigatus* and ARAF across soil and compost substrates, as well among the environment types, was also analyzed using Pearson χ^2^ test or Fisher exact test as appropriate. To determine which environmental variables could predict the ARAF culture growth in soil samples, we applied generalized linear models using the *lme4* package (v1.1.37) and penalized logistic regression with L1 regularization (Lasso) via the *glmnet* package (v4.1.10). The binary outcome variable was ARAF culture status (positive/negative), and the predictor variables included environment type, sample type, and exposure to generic triazole DMI fungicides. All statistical analyses and visualizations were conducted in R (version 4.4.2).

## RESULTS

### ARAF and *cyp51A* TR Characterization in Air Samples

**Figure 1:**
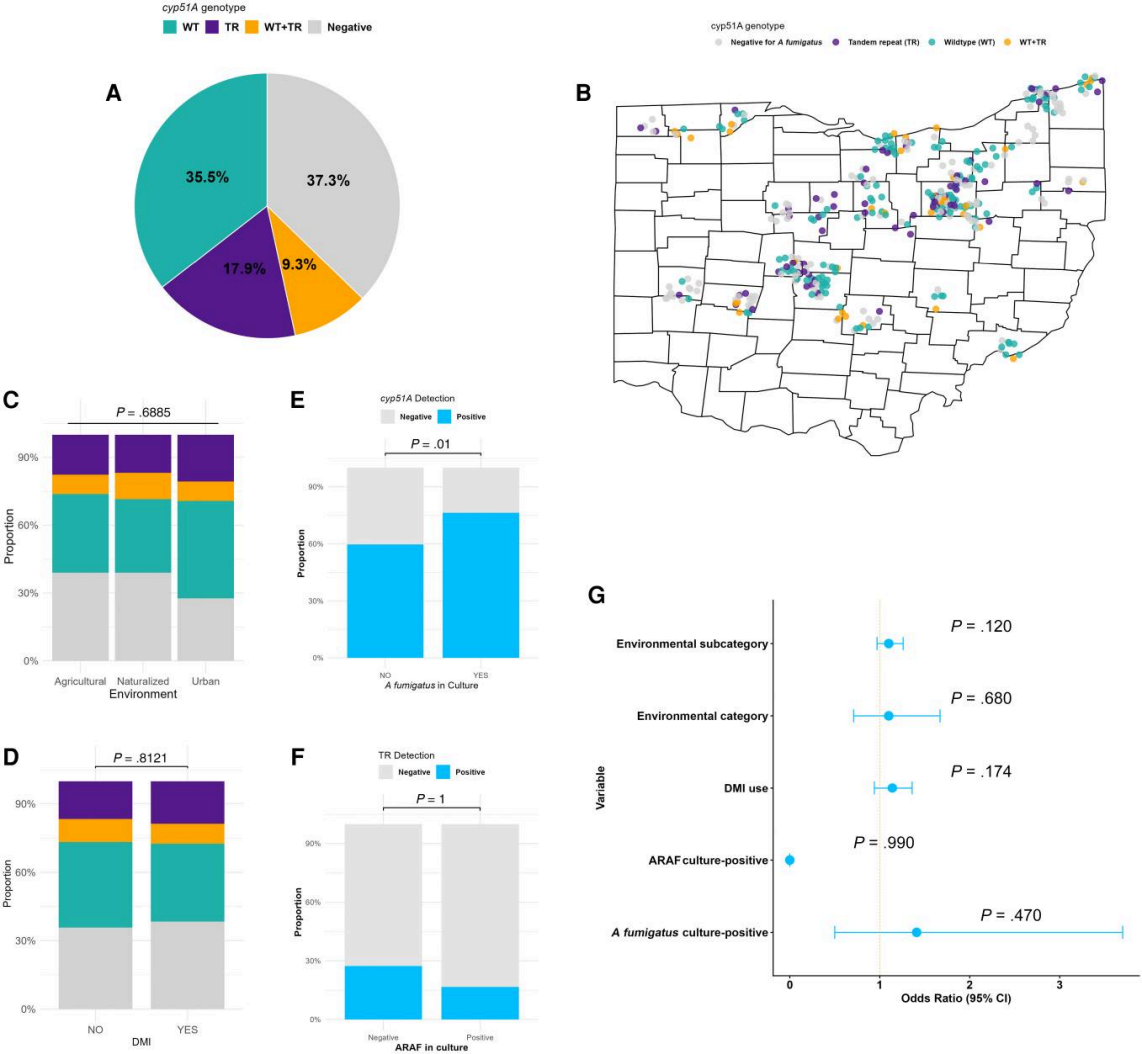
Prevalence of triazole resistance-associated *cyp51*A tandem repeat genotypes in airborne spore sample DNA extracts directly by nested PCR. *A*, Proportion of *cyp51*A genotypes, wild-type (WT), tandem repeat (TR), and mixed genotype (WT + TR) in airborne spore samples. *B*, Distribution of *cyp51*A genotypes across sampling locations in Ohio. *C*, Proportional distribution of *cyp51*A genotypes among environment types. *D*, Proportional distrubution of *cyp51*A geneotypes among demethylase inhibitor (DMI) fungicide-exposed or nonexposed sites. *E*, Association between *A fumigatus* detection in culture and *cyp51*A detection by nested PCR. *F*, Association between azole-resistant *A fumigatus* (ARAF) detection in culture and TR detection by nested PCR. *G*, Forest plot depicting the odds ratio, 95% confidence intervals, and *P* values calculated for independent variables assessed in a logistic regression model to predict *cyp51A* TR mutation in airborne spore samples.

A total of 411 air samples were collected from the agricultural (n = 244), urban (n = 58), naturalized (n = 95), and commercial (n = 14) environments (Table 1). Using nested PCR on samples originating from the agricultural, urban, and naturalized environments only (n = 397), more than one-third (n = 148 [37.3%]) of the samples tested negative for *A fumigatus* DNA, and the WT genotype was detected in 35.5% (n = 141) of the samples. The *cyp51A* TR mutations were identified in 17.9% (n = 71) of the samples and 9.3% (n = 37) of the samples showed presence of both WT and TR *cyp51A* genotypes (Figure 1*A*). The *cyp51A* TR mutation was noted in 82.4% (61/74) of the environments sampled across Ohio. *cyp51A* TR was noted in 26.2% (64/244) of samples from agricultural environments, 29.3% (17/58) from urban environments, and 28.4% (27/95) from naturalized environments. The *cyp51A* genotypes in the air samples were evenly distributed across Ohio (Figure 1*B*). Further*, cyp51A* genotypes were evenly distributed among different environment types (χ^2^ = 3.91, degrees of freedom [*df*] = 6, *P* = .69) as well as sampling sites with or without a DMI fungicide application (χ^2^ = 0.96, *df* = 3, *P* = .81) (Figure 1*C* and 1*D*). *Aspergillus fumigatus* was detected by culture in 18.5% (76/411) of samples, whereas nested PCR detected *A fumigatus* DNA in 62.7% (249/397) of the samples (McNemar χ^2^ = 146.8, *df* = 1, *P* < 2.2 × 10^−14^). Detection of *A fumigatus cyp51A* by nested PCR assay was significantly associated with a positive *A fumigatus* culture (χ^2^ = 6.3, *df* = 1, *P* = .01; Figure 1*E*). The phenotypic detection of ARAF by culture (6/397 [1.5%]) was lower than *cyp51A* TR genotype detection by nested PCR (108/397 [27.2%]), although the difference was not statistically significant (Fisher exact test, *P* = 1; Figure 1*F*). The generalized logistic regression model showed that TR presence in air samples was independent of *A fumigatus* culture positivity (odds ratio [OR], 1.4 [95% CI, .5–3.7], *P* = .47), ARAF culture positivity (*P* = .99), environmental category (OR, 1.1 [95% CI, .71–1.67], *P* = .68), environmental subcategory (OR, 1.1 [95% CI, .97–1.26], *P* = .12), or DMI use (OR, 1.1 [95% CI, .94–1.36], *P* = .174) (Figure 1*G*).

**Table 1.** Adjusted, Main Effects, and Lasso-Regularized Generalized Linear Models of Environmental Variables Predictive of Azole-Resistant *Aspergillus fumigatus*

Model	Variable	Odds Ratio (95% CI)	*P* Value
Adjusted model	Sample type		
	Compost	5.8 (1.55–25)	.**012**
	Triazole DMI exposure		
	Propiconazole	8.38 (1.36–162)	.054
	Tebuconazole	2.23 (.03–108)	.691
	Mefentrifluconazole	2.21 (.04–93.6)	.666
	Fenbuconazole	9.48 (.15–5120)	.342
	Environment		
	Agricultural	0.71 (.09–10.6)	.764
	Naturalized	1.90 (.05–95.8)	.71
Main effects model	Sample type		
	Compost	5.2 (1.52–18.9)	.**009**
	Triazole DMI exposure		
	Propiconazole	6.2 (1.34–44.3)	.**03**
	Tebuconazole	2.8 (.14–46)	.49
	Mefentrifluconazole	2.2 (.1–36.3)	.59
	Fenbuconazole	6.3 (.13–537)	.35
Lasso regularized model	Sample type		
	Compost	6.1 (1.8–22.5)	.**004**
	Triazole DMI exposure		
	Propiconazole	7.5 (1.6–53.5)	.**02**
	Tebuconazole	3.8 (.2–66.5)	.39
	Mefentrifluconazole	1.9 (.1–32.7)	.69
	Fenbuconazole	10.5 (.2–990)	.25

*P* values shown in bold font represent statistically significant predictors of ARAF.

Abbreviations: CI, confidence interval; DMI, demethylase inhibitor.

### ARAF and TR Characterization in Soil and Compost Samples

**Figure 2.**
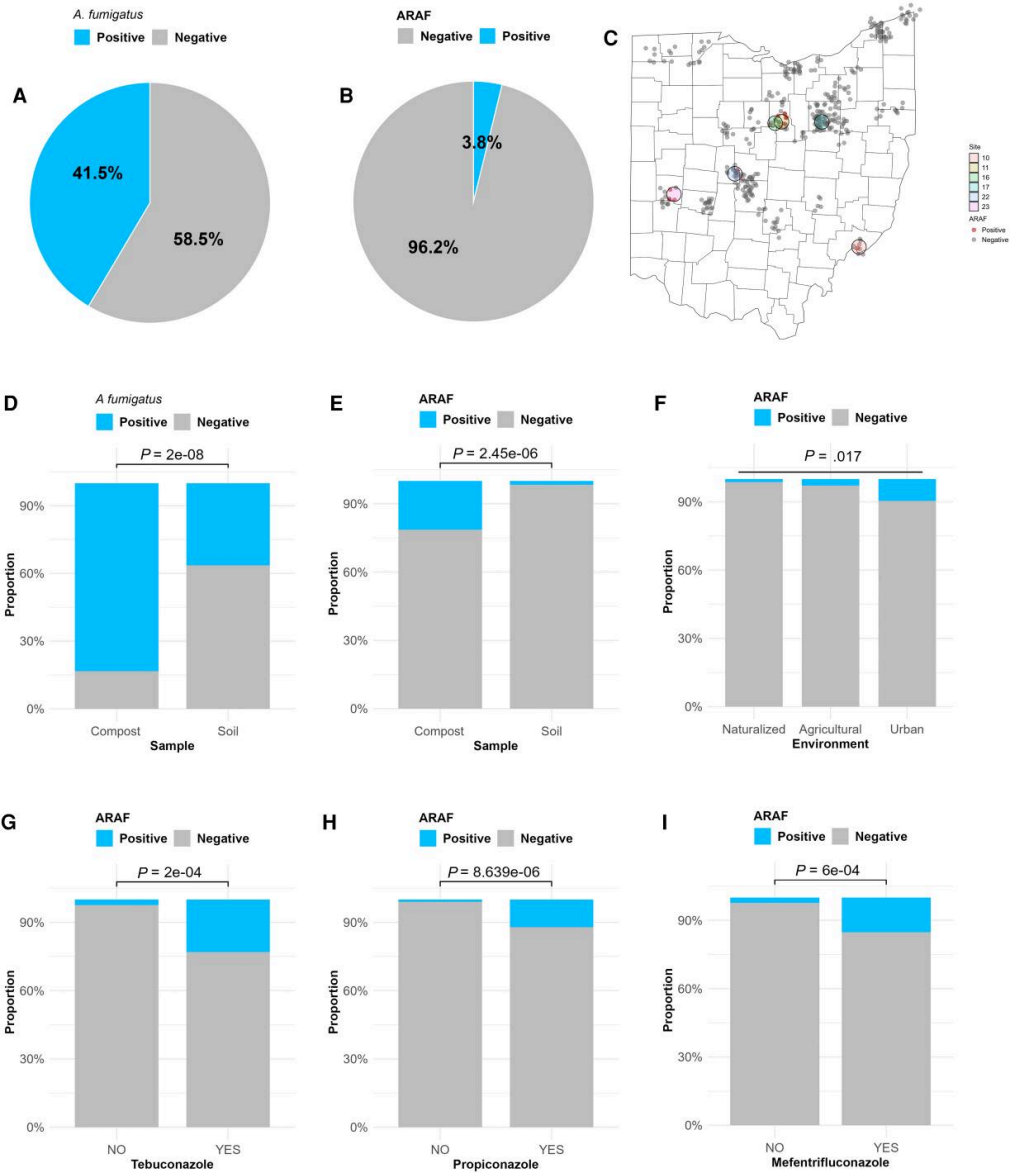
Prevalence of azole-resistant *A fumigatus* (ARAF) in soil and compost samples determined by culture. *A*, *A fumigatus* burden in soil/compost samples. *B*, ARAF burden in soil and compost samples. *C*, Distribution of ARAF-positive and negative samples across Ohio (circled data points indicate sampling sites that tested positive for ARAF). *D*, Distribution differences of *A fumigatus* in soil and compost. *E*, Distribution differences of ARAF in soil and compost. *F*, Distribution differences in ARAF across environment category. *G–I*, Distribution differences in ARAF across three triazole demethylase inhibitor fungicides.

Among 352 replicate soil and 42 compost samples, *A fumigatus* growth was noted in 41.4% (163/394) of samples (Figure 2*A*). The overall prevalence of ARAF was estimated at 3.8% (15/394) among all collected samples and 9.2% (15/163) among *A fumigatus* culture-positive samples (Figure 2*B*). The ARAF-positive samples included 9 compost samples and 6 soil samples originating from 6 sampling sites across Ohio (Figure 2*C*). *Aspergillus fumigatus* culture positivity was higher in compost samples compared to soil samples (83.3% vs 36.4%; χ^2^ = 32.04, *df* = 1, *P* = 1.51 × 10^−8^) (Figure 2*D*). ARAF recovery was significantly associated with compost compared to soil samples (21.4% vs 1.7%; Fisher exact test, *P* = 2.4 × 10^−6^) (Figure 2*E*). The ARAF-positive soil or compost sampling sites included a strawberry farm (3/5 samples ARAF), a cut flower farm (3/5 samples ARAF), a golf course (6/14 samples ARAF), an apple orchard (1/8 ARAF), a city public park (1/5 ARAF), and a wildlife area (1/5 ARAF). The presence of ARAF was more common in urban (9.5%) and agricultural environments (2.8%) compared to naturalized settings (1.4%), and the difference was statistically significant (Fisher exact test, *P* = .02) (Figure 2*F*). Exposure and nonexposure to DMIs was recorded in 5.3% and 1.3% of ARAF samples, respectively, and the association of overall DMI exposure with recovery of ARAF was statistically nonsignificant (χ^2^ = 3.1, *df* = 1, *P* = .08). However, exposure to specific triazole DMIs was significantly associated with ARAF recovery, including tebuconazole (23% vs 2.4%; Fisher exact test, *P* = 1.55 × 10^−4^), propiconazole (12.1% vs 1%; Fisher exact test, *P* = 8.6 × 10^−6^), and mefentrifluconazole (15.2% vs 2.3%; Fisher exact test, *P* = 6 × 10^−4^) (Figure 2*G* and 2*I*).

The surveillance data are publicly available on a tableau dashboard on the Ohio State University website (https://go.osu.edu/azole_resistant_araf).

Nested PCR analysis of 187 soil samples from 71 environments showed that 37.4% (70/187) were WT, 17.6% (33/187) were TR, and 5.9% (11/187) were a mixture of WT and TR. A total of 39.03% (73/187) of the soil samples were negative for *A fumigatus* target DNA amplification.

### Generalized Linear Models for ARAF Prediction in Soil Samples

In the initial generalized linear model, compost was the only significant predictor of ARAF positivity (OR, 33.8 [95% CI, 3.4–979.5], *P* = .009). Model performance was evaluated using Tjur's *R*^2^ (0.48) and receiver operating characteristic curve (ROC) analysis, yielding an area under the curve (AUC) of 0.98 (Supplementary Figure 1*A*). To assess the stability of this model, a stepwise reduction of environmental variables was performed. After excluding environmental subcategory, the predictive accuracy of the model remained stable with compost as the significant predictor and propiconazole showing a marginal association with ARAF (adjusted model; Tjur's *R*^2^ = 0.28; AUC = 0.89) (Table 1 and Supplementary Figure 1*B*). Further reduction of the model by excluding the environment category variable resulted in propiconazole achieving statistical significance as an independent predictor (main effects model; Tjur's *R*^2^ = 0.27, AUC = 0.88) (Table 1 and Supplementary Figure 1*C*).

To explore the most conservative predictor variables, we applied Lasso regularization with variables including sample type, exposure to triazole DMIs, and environment type. Lasso regularization reaffirmed compost and propiconazole exposure as independent risk factors for ARAF (Table 1). Predictive performance of the Lasso model was assessed using McFadden's pseudo-*R*^2^ (0.32) and ROC analysis (AUC, 0.85) (Supplementary Figure 1*D*).

### *cyp51A* Mutation Analysis of ARAF Isolates

Using the propiconazole-amended replica plate method, a total of 72 putative ARAF isolates were purified from 15 soil and compost samples and 8 air samples originating from 12 sampling sites of which 50% were exposed to DMI fungicides (Table 2). Of these 72 ARAF isolates, 50% of the isolates (36/72) harbored the TR_34_/L98H environmental resistance signature, whereas 43.05% (31/72) exhibited the TR_46_/Y121F/T289A marker, including 3 isolates (MLI-3527, MLI-3533, MLI-3542) with (TR_46_)3/Y121F/T289A/G448S variant. Two isolates (MLI-3772, MLI-3788) carried an indel mutation of 130 bp *cyp51A* promoter (−330 bp upstream relative to start codon), resulting in duplication of the sterol regulatory element binding site (SRE) (ATCACGCGGT), CCAAT binding complex site (CBC) (CGAAT), and heme activator protein (HapX) binding site (TTACTAA) (Supplementary Data 1). One isolate (MLI-3796) also harbored the indel with duplicated SRE, CBC, and HapX sites; however, the SRE site exhibited variation in 2 bases at the 3′ end of the motif, whereas 1 isolate (MLI-3788) exhibited no alteration in *cyp51*A.

**Table 2.** Minimum Inhibitory Concentrations and Environmental *cyp51A* Signature Mutations in Azole-Resistant *Aspergillus fumigatus* Isolates Recovered From Air, Soil, and Compost Samples

Isolate ID	Sample ID	Environment Subcategory	Sample	MIC, µg/mL					*cyp*51A AMRD	DMI Exposure
				PPZ	TBZ	ITZ	PSZ	VRZ		
MLI-1250	1a	Open windrow compost	Air	>64	8	>32	1	1	TR_34_/L98H	Not known
MLI-1251	1a	Open windrow compost	Air	>64	16	>32	1	1	TR_34_/L98H	
MLI-1252	1a	Open windrow compost	Air	>64	32	>32	1	1	TR_34_/L98H	
MLI-1256	5d	Open windrow compost	Air	>64	32	>32	1	1	TR_34_/L98H	
MLI-3485	AFRI-38	Cut flower farm	Air	>64	64	>32	1	32	TR_46_/Y121F/T289A	Tetraconazole
MLI-3486	AFRI-38	Cut flower farm	Air	>64	64	>32	1	32	TR_46_/Y121F/T289A	
MLI-3487	AFRI-38	Cut flower farm	Air	>64	64	>32	1	2	TR_34_/L98H	
MLI-3488	SS-19	Apple orchard	Soil	32	32	>32	1	2	TR_34_/L98H	Mefentrifluconazole, fenbuconazole
MLI-3489	SS-19	Apple orchard	Soil	32	32	>32	0.5	2	TR_34_/L98H	
MLI-3490	SS-19	Apple orchard	Soil	>64	32	>32	1	4	TR_34_/L98H	
MLI-3494	SS-20	Apple orchard	Compost	>64	64	>32	0.5	32	TR_34_/L98H	
MLI-3495	SS-20	Apple orchard	Compost	>64	16	>32	0.5	2	TR_34_/L98H	
MLI-3496	SS-20	Apple orchard	Compost	32	16	>32	0.5	32	TR_34_/L98H	
MLI-3497	SS-20	Apple orchard	Compost	>64	64	>32	0.5	>32	Not analyzed	
MLI-3498	SS-20	Apple orchard	Compost	>64	>64	>32	0.5	>32	TR_46_/Y121F/T289A	
MLI-3499	SS-20	Apple orchard	Compost	>64	64	>32	0.5	32	TR_46_/Y121F/T289A	
MLI-3500	SS-20	Apple orchard	Compost	>64	64	>32	0.5	32	TR_46_/Y121F/T289A	
MLI-3501	SS-20	Apple orchard	Compost	>64	>64	0.25	0.5	>32	TR_46_/Y121F/T289A	
MLI-3522	AFRI-55	Golf course	Air	64	32	>32	0.25	32	TR_34_/L98H	Mefentrifluconazole, tebuconazole, propiconazole
MLI-3527	SS-39	Golf course	Green waste compost	>64	>64	>32	1	>32	TR_46_^3/^Y121F/M172I/T289A/G448S	
MLI-3528	SS-39	Golf course	Green waste compost	>64	64	4	2	4	TR_34_/L98H	
MLI-3529	SS-40	Golf course	Green waste compost	64	64	1	1	32	TR_46_/Y121F/T289A	
MLI-3530	SS-40	Golf course	Green waste compost	>64	>64	>16	2	16	TR_34_/L98H	
MLI-3531	SS-40	Golf course	Green waste compost	>64	>64	>16	2	16	TR_34_/L98H	
MLI-3532	SS-40	Golf course	Green waste compost	>64	64	4	2	4	TR_34_/L98H	
MLI-3533	SS-40	Golf course	Green waste compost	>64	>64	2	2	>32	TR_46_^3/^Y121F/M172I/T289A/G448S	
MLI-3534	SS-40	Golf course	Green waste compost	>64	>64	4	2	>32	TR_46_/Y121F/T289A	
MLI-3535	SS-40	Golf course	Green waste compost	>64	64	8	2	1	TR_34_/L98H	
MLI-3536	SS-40	Golf course	Green waste compost	>64	64	8	2	1	TR_34_/L98H	
MLI-3537	SS-41	Golf course	Green waste compost	>64	>64	4	2	>32	TR_34_/L98H	
MLI-3538	SS-41	Golf course	Green waste compost	>64	>64	4	2	>32	TR_46_/Y121F/T289A	
MLI-3539	SS-41	Golf course	Green waste compost	>64	>64	1	1	32	TR_46_/Y121F/T289A	
MLI-3540	SS-41	Golf course	Green waste compost	>64	>64	4	2	>32	TR_46_/Y121F/T289A	
MLI-3541	SS-41	Golf course	Green waste compost	>64	64	8	2	1	TR_34_/L98H	
MLI-3542	SS-41	Golf course	Green waste compost	>64	>64	1	2	>32	TR_46_^3/^Y121F/M172I/T289A/G448S	
MLI-3543	SS-41	Golf course	Green waste compost	>64	64	8	2	1	TR_34_/L98H	
MLI-3544	SS-42	Golf course	Green waste compost	>64	64	8	2	1	TR_34_/L98H	
MLI-3545	SS-42	Golf course	Green waste compost	>64	>64	1	1	32	TR_46_/Y121F/T289A	
MLI-3546	SS-42	Golf course	Green waste compost	>64	64	4	2	1	TR_34_/L98H	
MLI-3549	SS-42	Golf course	Green waste compost	>64	64	4	2	1	TR_34_/L98H	
MLI-3550	SS-42	Golf course	Green waste compost	>64	>64	2	2	>32	TR_46_/Y121F/T289A	
MLI-3551	SS-42	Golf course	Green waste compost	>64	>64	2	2	>32	TR_46_/Y121F/T289A	
MLI-3552	SS-42	Golf course	Green waste compost	>64	>64	>16	2	8	TR_34_/L98H	
MLI-3553	SS-42	Golf course	Green waste compost	>64	>64	2	1	>32	TR_46_/Y121F/T289A	
MLI-3554	SS-44	Golf course	Green waste compost	>64	32	>16	2	1	TR_34_/L98H	
MLI-3555	SS-44	Golf course	Wood chipping compost	>64	>64	2	2	>32	TR_46_/Y121F/T289A	
MLI-3556	SS-44	Golf course	Wood chipping compost	>64	32	>16	2	1	TR_34_/L98H	
MLI-3557	SS-44	Golf course	Wood chipping compost	>64	32	>16	2	1	TR_34_/L98H	
MLI-3558	SS-45	Golf course	Green waste compost	>64	32	>16	2	1	TR_34_/L98H	
MLI-3596	SS-60	Public park	Soil	>64	>64	1	1	>32	TR_46_/Y121F/T289A	Not known
MLI-3679	SS-75	Marshy land	Soil	>64	16	>32	2	1	TR_34_/L98H	Not known
MLI-3772	AFRI-314	Cut flower farm	Air	16	32	2	0.25	2	Indel 130	Propiconazole
MLI-3788	AFRI-321	Cut flower farm	Air	8	16	1	0.25	2	Indel 130	No
MLI-3796	AFRI-325	Vegetable farm	Air	32	8	0.5	0.12	1	Indel 130	No^a^
MLI-3869	AFRI-348	Flower farm	Air	32	16	0.5	0.12	1	Wild-type	No
MLI-3905	SS-97	Cut flower farm	Compost	>64	64	2	2	>32	TR_46_/Y121F/T289A	Propiconazole
MLI-3906	SS-97	Cut flower farm	Compost	>64	>64	2	2	>32	TR_46_/Y121F/T289A	
MLI-3907	SS-97	Cut flower farm	Compost	>64	>64	2	2	>32	TR_46_/Y121F/T289A	
MLI-3908	SS-98	Cut flower farm	Compost	>64	64	2	1	>32	TR_46_/Y121F/T289A	
MLI-3909	SS-98	Cut flower farm	Compost	>64	64	2	1	>32	TR_46_/Y121F/T289A	
MLI-3910	SS-99	Cut flower farm	Compost	>64	>64	2	1	>32	TR_46_/Y121F/T289A	
MLI-3911	SS-99	Cut flower farm	Compost	>64	>64	2	1	>32	TR_46_/Y121F/T289A	
MLI-3912	SS-99	Cut flower farm	Compost	>64	>64	4	2	>32	TR_46_/Y121F/T289A	
MLI-3913	SS-103	Berry farm	Soil	>64	>64	2	2	>32	TR_46_/Y121F/T289A	Propiconazole
MLI-3914	SS-103	Berry farm	Soil	>64	>64	2	2	>32	TR_46_/Y121F/T289A	
MLI-3915	SS-103	Berry farm	Soil	>64	>64	2	2	>32	TR_46_/Y121F/T289A	
MLI-3916	SS-104	Berry farm	Soil	>64	32	>32	2	1	TR_34_/L98H	
MLI-3917	SS-104	Berry farm	Soil	>64	64	>32	2	2	TR_34_/L98H	
MLI-3918	SS-104	Berry farm	Soil	>64	64	>32	2	4	TR_34_/L98H	
MLI-3919	SS-106	Berry farm	Soil	>64	64	>32	2	4	TR_34_/L98H	
MLI-3920	SS-106	Berry farm	Soil	>64	64	>32	2	2	TR_34_/L98H	
MLI-3921	SS-106	Berry farm	Soil	>64	64	>32	2	4	TR_34_/L98H	

Abbreviations: AMRD, antimicrobial resistance determinant; DMI, demethylase inhibitor; ITZ, itraconazole; PPZ, propiconazole; PSZ, posaconazole; TBZ, tebuconazole; VRZ, voriconazole.

^a^DMI used in 2023.

### Relationship Between Phenotypic and Genotypic Resistance to Triazoles

**Figure 3.**
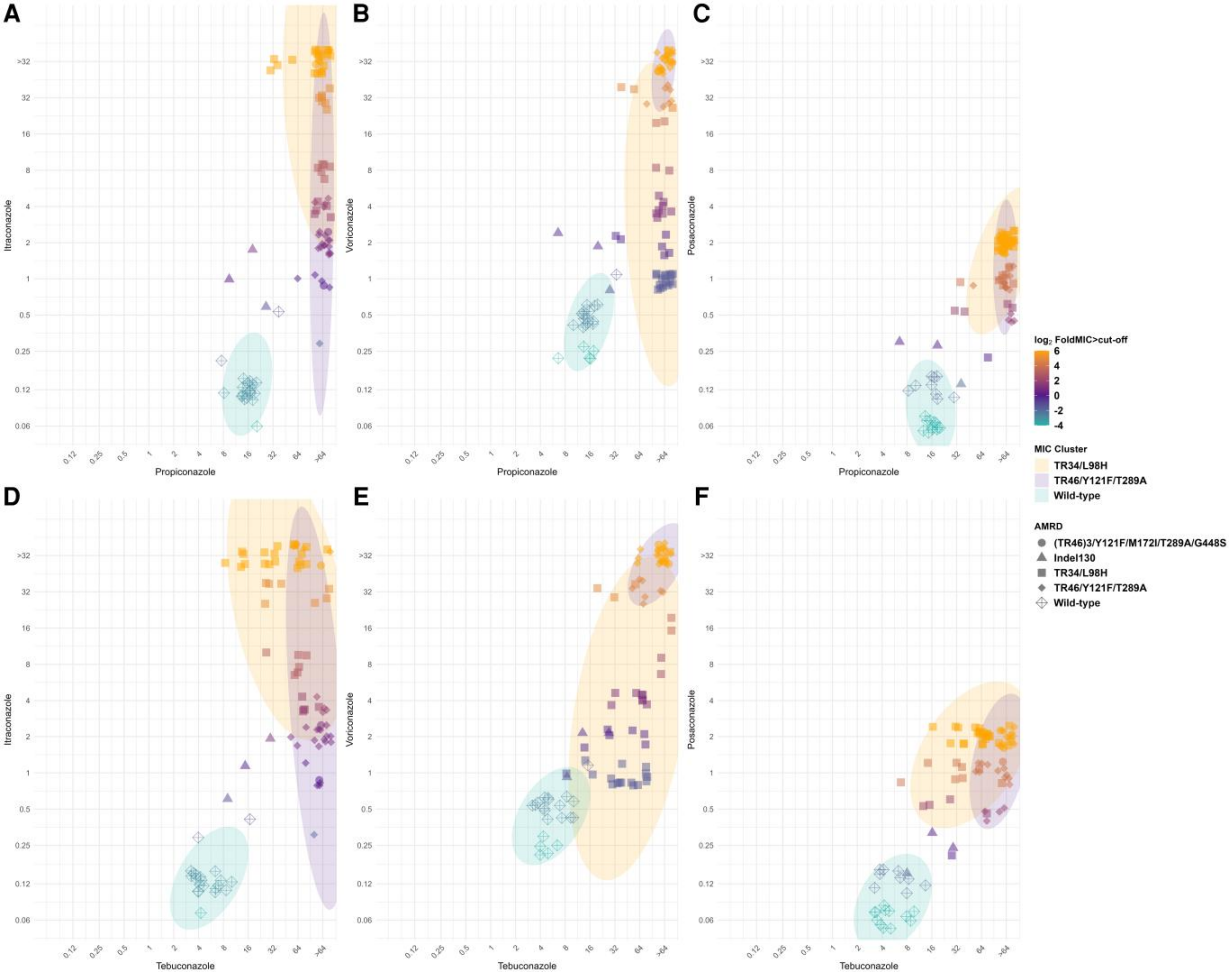
Relationship between minimum inhibitory concentration (MIC) distributions of triazole demethylase inhibtor fungicides (x-axis) and medical triazoles (y-axis) stratified by *cyp51*A antimicrobial resistant determinant (AMRD). *A–C*, Propiconazole versus medical triazoles. *D–F*, Tebuconazole versus medical triazoles. The color scale in AMRD shapes represents log_2-_fold MIC of itraconazole, voriconazole, and posaconazole above the CLSI epidemiological cutoff values. The ellipses show MIC clusters of *cyp51A* AMRD based on 95% confidence intervals (MIC of MLI3497 presented in Table 2 is not included in the plots).

Broadly distinct MIC distribution patterns were observed among *A fumigatus* isolates when stratified by *cyp51A* genotype (Figure 3). Of the 21 WT isolates, 20 isolates exhibited propiconazole MICs in the range of 8–16 µg/mL. All these isolates were susceptible to clinical triazoles, exhibiting MICs 2 to 4 log_2_-fold below the itraconazole breakpoint, 1 to 2 log_2_-fold lower than the voriconazole breakpoint, and 2 to 3 log_2_-fold below the posaconazole breakpoint (Figure 3*A*–*C*, Supplementary Table 2). In contrast, isolates carrying a TR_34_ or TR_46_ mutation exhibited propiconazole MIC ≥32 µg/mL. For TR_34_ isolates, MICs against clinical triazoles ranged from 2 to 6 log_2_-fold above the itraconazole breakpoint, 0 to 6 log_2_-fold above the voriconazole breakpoint, and −1 to 4 log_2_-fold above the posaconazole breakpoint. For TR_46_ isolates, MICs were −2 to 6, 5–6, and 0–2 log_2_-fold above the breakpoints for itraconazole, voriconazole, and posaconazole, respectively. For WT isolates, the MICs of tebuconazole were ≤8 µg/mL in 20 of 21 isolates, whereas 66 of 67 isolates with a TR mutation exhibited tebuconazole MICs ≥16 µg/mL (Figure 3*D*–*F*, Supplementary Table 2).

Isolates harboring the TR_34_/L98H mutation exhibited elevated itraconazole MICs (≥4 µg/mL) compared to those with the TR_46_/Y121F/T289A mutation in which the majority (21/31 [67.7%]) exhibited itraconazole MIC ≤2 µg/mL (Figure 3*A* and 3*D*). In contrast, the voriconazole MICs displayed an inverse trend with TR_46_/Y121F/T289A isolates demonstrating high-level resistance (MICs ≥32 µg/mL), while TR_34_/L98H isolates mostly clustered (31/38 [81.6%]) within the 1–4 µg/mL range (Figure 3*B* and 3*E*). This bimodal MIC distribution was not observed in posaconazole, where both TR_34_/L98H and TR_46_/Y121F/T289A isolates exhibited variable MICs above the established resistance threshold (Figure 3*C* and 3*F*). Two of the 3 isolates with the TR_46_^3^/Y121F/T289A/G448S mutation (MLI-3527, MLI-3533, MLI-3542) exhibited high-level pan-azole resistance (Table 2). Two isolates with an indel 130 *cyp51A* promoter mutation (MLI-3772 and MLI-3788) exhibited 1 log_2_fold higher MIC for voriconazole above the epidemiological cutoff value, whereas 1 isolate (MLI-3796) was susceptible to all 3 clinical triazoles. One isolate with WT *cyp51A* (MLI-3869), exhibited moderately elevated MIC for propiconazole (32 µg/mL), and tebuconazole (16 µg/mL); however, this isolate was susceptible to clinical triazoles (Table 2 and Supplementary Table 2).

## DISCUSSION

Environmental surveillance studies for the prevalence of ARAF in the US remain limited compared to Europe, making it difficult to accurately estimate its prevalence in local environments, despite multiple reports of ARAF isolation in healthcare settings in the country [22]. This study provides a comprehensive environmental surveillance of ARAF across diverse ecological settings in Ohio. By integrating air, soil, and compost sampling with both molecular and culture-based approaches, we demonstrate that ARAF is widespread, genetically diverse, and environmentally entrenched beyond traditional agricultural environments. This study also suggests that agricultural settings, in general, function as cold spots of ARAF, and the use of DMI fungicides in general farming may not necessarily pose a major public health risk. Instead, deployment of these compounds in urban and commercial environments may play a more significant role in contributing to the environmental ARAF burden.

To overcome the low yield of fungal culture and phenotypic detection of ARAF, environmental DNA samples may be directly probed for *cyp51A* TR. Our findings underscore the higher detection of TR directly from environmental DNA samples by nested PCR targeting the *cyp51A* promoter compared to phenotypic detection of ARAF by culture. Although surveillance of ARAF mostly relies on culture, similar observations of low detection rate of ARAF by culture compared to detection of resistance mutations independent of culture have been noted in clinical specimens [32–34]. These reports suggest that culture-based methods may underestimate fungal detection and resistance prevalence. On the contrary, while culture offers the advantage of capturing only viable resistant fungal propagules, direct molecular detection of ARAF from clinical or environmental DNA samples can be confounded or overestimated by nonviability of fungal elements, cross-reactivity, and other inherent limitations of PCR-based methods [35]. Therefore, in the absence of a reliable reference method, it is difficult to estimate the true burden of ARAF in the environment.

Although molecular assays are generally more sensitive than culture, the detection of resistance mutations in a single-copy *cyp51A* gene may be less sensitive compared to *A fumigatus* species detection using multicopy targets such as 28S rRNA gene [15]. These results support integrating molecular methods with conventional methods for accurate surveillance, as reliance on culture alone may fail to capture the full extent of resistant spore circulation in the environment.

ARAF was recovered from 16% of the environments sampled, with TR_34_/L98H and TR_46_/Y121F/T289A mutations occurring at nearly equal frequencies. Soil and compost analyses emphasized the importance of diverse environmental reservoirs in the emergence and persistence of ARAF. Although, the broad category of DMI exposure failed to reach statistical significance, univariate analysis indicated strong associations of specific DMI triazoles (tebuconazole, propiconazole, and mefentrifluconazole) to recovery of ARAF among soil/compost samples. Compost and propiconazole emerged as predictors of ARAF positivity associated with higher odds of ARAF and consistent significance across both generalized logistic regression and Lasso modeling. Although our generalized linear models were constrained by effective sample size, Lasso regularization ensured a conservative selection of predictors. Nevertheless, these modeling approaches lay the groundwork for broader environmental risk assessments of ARAF. The elevated prevalence of ARAF in compost compared to soil (21.4% vs 1.7%) further underscores the role of composting environments as “hotspots” for resistance selection and amplification, likely due to a combination of elevated temperatures, organic matter enrichment, and possible residual fungicide exposure [15]. Similar findings of low prevalence of ARAF in agricultural soils have been reported in the US and other countries [36, 37]. Ohio has at least 397 commercial open windrow composting facilities, and periodic turning of compost windrows has the potential to lead to wide dispersal of ARAF (https://geo.epa.ohio.gov/portal/apps/experiencebuilder/experience/?id=e8e3e42ec4854ee3802cb4c41fe9614c). For instance, in this study, TR_34_/L98H was identified in 36.4% of isolates recovered from air samples (n = 14) actively collected during the turning of the windrows at a commercial composting facility in Ohio. Amenity turf grasses remain under intensive management with fungicides, including DMIs such as tebuconazole, propiconazole, myclobutanil, triadimefon, and mefentrifluconazole, due to frequent outbreaks of fungal diseases [17]. We detected a high burden of *A fumigatus* in composting cull piles derived from green waste at a golf course. Although soil samples from the fairway and greens from the course tested negative for ARAF, 86% of composting grass clipping samples tested positive for ARAF with the presence of mixed genotypes of TR_34_/L98H, TR_46_/Y121F/T289A, and TR_46_^3^/Y121F/M172I/T289A/G448S mutations. These observations suggest that resistant genotypes, once established, spread broadly through airborne dissemination rather than persisting solely at local sites, further supporting previous research that compost-mediated resistance is an important contributor to community-level exposure [30]. The overwhelming presence of *A fumigatus* in compost and green waste coupled with the high likelihood of ARAF poses a significant public health risk, especially for people with altered immune function.

We noted a clear cross-resistance pattern among the DMI fungicides propiconazole and tebuconazole with medical triazoles, suggesting that these isolates may have evolved resistance in response to collateral exposure to DMI triazoles in the environment. Further, distinct distributions of low-level and high-level resistance were noted for itraconazole and voriconazole in relation to *cyp51A* mutation. TR_34_/L98H usually confer a high-level itraconazole resistance (MIC ≥16 µg/mL), whereas those with TR_46_/Y121F/T289A confer high-level resistance to voriconazole and isavuconazole [38]. Clinically, this differential triazole susceptibility of TR_34_/L98H and TR_46_/Y121F/T289A mutations has direct therapeutic implications. While both mutations lead to reduced triazole susceptibility, TR_46_/Y121F/T289A and its variant, TR_46_^3^/Y121F/T289A/G448S, are notably highly resistant to voriconazole, presenting a greater challenge for clinical management.

We evaluated cross-resistance between triazole DMIs, specifically, tebuconazole and propiconazole, with clinical triazoles as they are the most commonly used DMIs in the US [39]. Although imidazole DMIs were also reported in our surveillance, there is limited clarity on whether TR_34_/L98H and TR_46_/Y121F/T289A genotypes are selected only against triazole DMIs or imidazole DMIs as well. Based on the literature, TR genotypes appear to exhibit elevated MICs to imidazole DMIs, whereas isolates with WT *cyp51A* show higher sensitivity [40].

Although TR_34_/L98H mutation dominates globally, 43% of the isolates analyzed for *cyp51A* in our study harbored TR_46_/Y121F/T289A or its variant, TR_46_^3^/Y121F/T289A/G448S. A previous study from the US has also reported a higher proportion of TR_46_/Y121F/T289A in ARAF isolates recovered from soil, plant debris, and compost samples [23]. The detection of the TR_46_^3^/Y121F/T289A/G448S variant across Europe and the US is of particular concern, as this mutation confers high-level pan-azole resistance, further narrowing treatment options [41, 42]. These data underscore the urgent need for routine genotyping of clinical and environmental isolates to guide antifungal therapy.

Although *cyp51A* mutations are the major driver of ARAF, resistance mediated by mechanisms other than *cyp51A* mutations have been increasingly reported [43, 44]. Three isolates exhibited moderately reduced susceptibility and harbored a 130-bp indel mutation in the *cyp51A* promoter region. Notably, the indel encompassed repeat units of both TR_34_ and TR_46_, which are characteristic of the TR_34_/L98H and TR_46_/Y121F/T289A genotypes. However, while TR mutations duplicate only the SRE- and CBC- binding sites, 130 bp indel resulted in duplication of all 3 key regulatory binding sites in the *cyp51A* promoter: SRE, CBC, and HapX. HapX is a component of the combinatorial repression complex that functions together with CBC [45, 46]. The 130 bp indel mutation likely enhances *cyp51A* transcription, and as accompanying missense mutations were not detected in the coding sequence, it appears to contribute to the observed moderate reduction in triazole sensitivity in those isolates. However, further mechanistic studies are required to elucidate the role of this unusual promoter mutation in ARAF, which were beyond the scope of the present surveillance study. In addition, 1 isolate with WT *cyp51A* also demonstrated moderately elevated MICs to agricultural DMIs, suggesting that non-*cyp51A*-mediated mechanisms such as efflux pump overexpression or off-target mutations may also be contributing to resistance. These findings highlight the ongoing evolution of azole resistance beyond the canonical TR_34_/L98H and TR_46_/Y121F/T289A mutations.

Notwithstanding the clinical implications of environmental ARAF, the findings of this study must be interpreted cautiously. Given the complexity of host (immune status, site of infection, and azole pharmacokinetics) and microbiological factors that can influence the outcome of triazole therapy, in vitro ARAF may not invariably lead to clinical treatment failure [47, 48]. However, a high environmental burden of ARAF significantly increases the risk of treatment-refractory *A fumigatus* infections in clinical settings.

In conclusion, this study enhances our understanding of environmental surveillance of ARAF in the US, with a focus on diverse environments across the Midwestern state of Ohio. We believe this study provides a broad framework for more comprehensive surveillance studies to identify the key environmental variables and potential hotspots of ARAF.

## Supplementary Material

ofag150_Supplementary_Data

## Supplementary Data

Supplementary materials are available at *Open Forum Infectious Diseases* online. Consisting of data provided by the authors to benefit the reader, the posted materials are not copyedited and are the sole responsibility of the authors, so questions or comments should be addressed to the corresponding author.
