# Supplementary material for: Azole Resistance in Aspergillus fumigatus From Diverse Environments in Ohio, United States, Is Primarily Driven by TR34/L98H and TR46/Y121F/T289A Environmental Signatures
Source: Open Forum Infect Dis. 2026 Apr 21;13(4):ofag150. doi: 10.1093/ofid/ofag150 (PMC13095377; doi:10.1093/ofid/ofag150)
Supplement: ofag150_Supplementary_Data [file ofag150_supplementary_data.zip › PAUL ET AL Supplementary Data S1.pdf]

a) Trimmed *cyp51*A promoter sequences of reference, *Af*293 and isolates with 130 bp indel

>*Af*293

GTTGTCTAGAATCACGCGGTCCGGATGTGTGCTGAGCCGAATGAAAGTTGCCTAATTACTAAGGT
GTAGTTCCAGCATACCATACACCCTAACTCATACTACGGTAGGTAGATCTACTTACCTATGAACCT
ATATTGGTAGGTAGGTGAATATAAAATACAGCATGGAACATGTTTTTCATTAGCTGGTCTCTCATTCG
TCCTTGTCCTAGGCCTTAAGGAATCCAGTATATGAAATAATCCCTCTTATCCATTTTCCTCTTATTCTT
TTTCATTTCCCTCATCACTGCAACTCTAATCCTCGGGCTCACCCTCCCTGTGTCTCCTCGAAATG

>MLI3772

GTTGTCTAGAATCACGCGGTCCGGATGTGTGCTGAGCCGAATGAAAGTTGCCTAATTACTAAGCT
GAATAATTTACACTGTTCTCCTCTAGAAAAAACTCATGAGTGAATAATCGCAGCACCACTTCAGA
GTTGTCTAGAATCACGCGGTCCGGATGTGTGCTGAGCCGAATGAAAGTTGCCTAATTACTAAGGT
GTAGTTCCAGCATACCATACACCCTAACTCATACTACGGTAGGTAGATCTACTTACCTATGAACCT
ATATTGGTAGGTAGGTGAATATAAAATACAGCATGGAACATGTTTTTCATTAGCTGGTCTCTCATTCG
TCCTTGTCCTAGGCCTTAAGGAATCCAGTATATGAAATAATCCCTCTTATCCATTTTCCTCCTATTCT
TTTTCATTTCCCTCATCACTGCAACTCTAATCCTCGGGCTCACCCTCCCTGTGTCTCCTCGAAATG

>MLI3788

GTTGTCTAGAATCACGCGGTCCGGATGTGTGCTGAGCCGAATGAAAGTTGCCTAATTACTAAGCT
GAATAATTTACACTGTTCTCCTCTAGAAAAAACTCATGAGTGAATAATCGCAGCACCACTTCAGA
GTTGTCTAGAATCACGCGGTCCGGATGTGTGCTGAGCCGAATGAAAGTTGCCTAATTACTAAGGT
GTAGTTCCAGCATACCATACACCCTAACTCATACTACGGTAGGTAGATCTACTTACCTATGAACCT
ATATTGGTAGGTAGGTGAATATAAAATACAGCATGGAACATGTTTTTCATTAGCTGGTCTCTCATTCG
TCCTTGTCCTAGGCCTTAAGGAATCCAGTATATGAAATAATCCCTCTTATCCATTTTCCTCCTATTCT
TTTTCATTTCCCTCATCACTGCAACTCTAATCCTCGGGCTCACCCTCCCTGTGTCTCCTCGAAATG

>MLI3796

GTTGTCTAGAATCACGCGGTCCGGATGTGTGCTGAGCCGAATGAAAGTTGCCTAATTACTAAGGT
GAATAATTTACACTGCTCTCCTCTAAAAAAAACTCATGAGTGAATAATCTCATCACCACTTCACATTT
GTCGAGAATCACGCGAACCGGATGTGTGCTGAGCCGAATGAAATTTGCCTAATTACTAATGTGTA
GTTCCTGCATACCATACACCCTAACTCATACTACGGTAGGTACATCTACTTACCTATGAACCTATAT
CGGTAGGTATGTGAATATAACAGACAGTATGGAACATGTTTTTCATTAGCTGGTCTCTCATTCGTCC

TTGTCCTAAGCCTTAAGGAATCCAGCATATGAAATAATCCCTCTTATACATTTTCCTCGTATTCTGTTT
CATTTCCCTCATCACTGCAACTCTAATCCTCGGGCTCACCCTCCCTGTGTCTCCTCTGAATG

Key:

Gray = Indel130

Green = Sterol regulatory element (SRE)-binding site

Turquoise = CCAAT complex-binding (CBC) site

Yellow = Heme activator protein (HapX- binding site

ATG = Start codon

b) Full *cyp51*A sequences of ARAF isolates with 130 bp indel (MLI33772, MLI3788, MLI3796) and ARAF isolate with *wildtype cyp51*A (MLI3869)

>MLI3772

CCCAAAGGGGGNAAGGAAAAGCACTCTGAATAATTTACACTGTTCTCCTCTAGAAAAACTCATGA
GTGAATAATCGCAGCACCACTTCAGAGTTGTCTAGAATCACGCGGTCCGGATGTGTGCTGAGCC
GAATGAAAGTTGCCTAATTACTAAGCTGAATAATTTACACTGTTCTCCTCTAGAAAAAACTCATGAGT
GAATAATCGCAGCACCACTTCAGAGTTGTCTAGAATCACGCGGTCCGGATGTGTGCTGAGCCGA
ATGAAAGTTGCCTAATTACTAAGGTGTAGTTCCAGCATACCATACACCCTAACTCATACTACGGTA
GGTAGATCTACTTACCTATGAACCTATATTGGTAGGTAGGTGAATATAAAATACAGCATGGAACATG
TTTTTCATTAGCTGGTCTCTCATTCGTCCTTGTCCTAGGCCTTAAGGAATCCAGTATATGAAATAATC
CCTCTTATCCATTTTCCTCCTATTCTTTTTCATTTCCCTCATCACTGCAACTCTAATCCTCGGGCTCA
CCCTCCCTGTGTCTCCTCGAAATGGTGCCGATGCTATGGCTTACGGCCTACATGGCCGTTGCG
GTGCTGACGGCAATCTTGCTCAATGTTGTTTATCAATTATTCTTTCGGCTTTGGAACCGAACAGAA
CCGCCAATGGTCTTTCATTGGGTCCCATTTCTGGGTAGTACCATCAGTTACGGGATTGATCCCTA
CAAGTTCTTCTTTGCGTGCAGAGAAAAGGCAAGTCTCAAGATTGTAGTTTGACATTCATTCCTGGG
CGCATTGCTGAGTATTGCTTTCTTAACCGGCAGTATGGCGATATCTTCACTTTTATACTGTTGGGTC
AAAAAACCACAGTCTACCTGGGCGTTCAGGGGAACGAGTTTATTCTCAACGGCAAGCTCAAGG
ATGTCAATGCGGAAGAGGTCTATAGTCCATTGACGACCCCCGTTTTCGGATCGGACGTGGTGTAT
GATTGTCCCAATTCCAAGCTGATGGAGCAGAAAAAGTTCATCAAGTACGGCTTGACTCAGTCTGC
GTTAGAGTCTCATGTGCCACTTATTGAGAAGGAGGTTTTGGACTATCTGCGCGATTCACCGAACTT
TCAAGGCTCGTCCGGCCGGATGGACATCTCTGCGGCAATGGCTGAGATTACCATTTTTACCGCT
GCTCGAGCCCTCCAAGGCCAGGAAGTTCGTTCCAAACTCACGGCTGAGTTCGCTGACCTCTAT
CATGACCTGGACAAGGGCTTTACTCCCATCAATTTTATGCTACCGTGGGCCCCATTGCCGCATA
ACAAGAAGCGAGATGCTGCTCATGCGCGCATGAGGTCAATCTACGTTGACATCATCAATCAGCG
CCGTCTTGACGGTGACAAGGACTCTCAGAAATCAGACATGATATGGAACCTGATGAACTGCACAT
ACAAAAACGGCCAGCAAGTGCCTGATAAAGAGATTGCGCACATGATGATAACCCTGTTGATGGC
TGGTCAGCATTCGTCTTCGTCCATCAGCGCCTGGATTATGCTGAGACTGGCCTCACAGCCAAAA
GTCCTCGAAGAGCTGTATCAGGAACAGCTGGCCAATCTTGGCCCCGCCGGGCCAGACGGCA
GTCTTCCTCCGCTCCAGTACAAGGATCTTGACAAACTTCCCTTCCATCAACATGTTATTCGTGAAA
CCTTACGGATTCACTCCTCTATTCACTCTATCATGCGCAAGGTGAAAAGCCCCTTGCCCGTTCCC
GGGACCCCTTACATGATTCCTCCCGGTCGCGTGCTCCTTGCTTCACCTGGAGTGACAGCCCTC
AGCGACGAACACTTCCCCAATGCTGGGTGCTGGGATCCCCATCGCTGGGAGAACCAGGCTAC
TAAGGAGCAGGAGAACGACGAGGTTGTCGACTACGGTTACGGCGCCGTCTCCAAGGGCACGT
CAAGTCCCTATCTTCCGTTTGGTGCTGGCCGACACCGCTGTATCGGCGAGAAATTCGCTTATGT
CAACCTTGGTGTGATTCTGGCGACCATTGTGCGCCACCTGCGACTTTCAACGTGGATGGAAAGA
AAGGAGTCCCTGAAACTGACTATTCATCCCTCTTTTCGGGCCCCATGAAGCCAAGCATCATCGG
CTGGGAGAAGCGGTCGAAAAACACATCCAAGTGAG

>MLI3788

CCCAAAGGGGGGAAAGGAAAACCTCTGAATAATTTACACTGTTCTCCTCTAGAAAAACTCATGAG
TGAATAATCGCAGCACCACTTCAGAGTTGTCTAGAATCACGCGGTCCGGATGTGTGCTGAGCCG
AATGAAAGTTGCCTAATTACTAAGCTGAATAATTTACACTGTTCTCCTCTAGAAAAAACTCATGAGTG
AATAATCGCAGCACCACTTCAGAGTTGTCTAGAATCACGCGGTCCGGATGTGTGCTGAGCCGAA
TGAAAGTTGCCTAATTACTAAGGTGTAGTTCCAGCATACCATACACCCTAACTCATACTACGGTAG
GTAGATCTACTTACCTATGAACCTATATTGGTAGGTAGGTGAATATAAAATACAGCATGGAACATGTT
TTTCATTAGCTGGTCTCTCATTCGTCCTTGTCCTAGGCCTTAAGGAATCCAGTATATGAAATAATCC
CTCTTATCCATTTTCCTCCTATTCTTTTTCATTTCCCTCATCACTGCAACTCTAATCCTCGGGCTCAC
CCTCCCTGTGTCTCCTCGAAATGGTGCCGATGCTATGGCTTACGGCCTACATGGCCGTTGCGGT
GCTGACGGCAATCTTGCTCAATGTTGTTTATCAATTATTCTTTCGGCTTTGGAACCGAACAGAACC
GCCAATGGTCTTTCATTGGGTCCCATTTCTGGGTAGTACCATCAGTTACGGGATTGATCCCTACAA
GTTCTTCTTTGCGTGCAGAGAAAAGGCAAGTCTCAAGATTGTAGTTTGACATTCATTCCTGGGCGC
ATTGCTGAGTATTGCTTTCTTAACCGGCAGTATGGCGATATCTTCACTTTTATACTGTTGGGTCAAAA
AACCACAGTCTACCTGGGCGTTCAGGGGAACGAGTTTATTCTCAACGGCAAGCTCAAGGATGTC
AATGCGGAAGAGGTCTATAGTCCATTGACGACCCCCGTTTTCGGATCGGACGTGGTGTATGATTG
TCCCAATTCCAAGCTGATGGAGCAGAAAAAGTTCATCAAGTACGGCTTGACTCAGTCTGCGTTAG
AGTCTCATGTGCCACTTATTGAGAAGGAGGTTTTGGACTATCTGCGCGATTCACCGAACTTTCAAG
GCTCGTCCGGCCGGATGGACATCTCTGCGGCAATGGCTGAGATTACCATTTTTACCGCTGCTCG
AGCCCTCCAAGGCCAGGAAGTTCGTTCCAAACTCACGGCTGAGTTCGCTGACCTCTATCATGA
CCTGGACAAGGGCTTTACTCCCATCAATTTTATGCTACCGTGGGCCCCATTGCCGCATAACAAG
AAGCGAGATGCTGCTCATGCGCGCATGAGGTCAATCTACGTTGACATCATCAATCAGCGCCGTC
TTGACGGTGACAAGGACTCTCAGAAATCAGACATGATATGGAACCTGATGAACTGCACATACAAA
AACGGCCAGCAAGTGCCTGATAAAGAGATTGCGCACATGATGATAACCCTGTTGATGGCTGGTC
AGCATTCGTCTTCGTCCATCAGCGCCTGGATTATGCTGAGACTGGCCTCACAGCCAAAAGTCCT
CGAAGAGCTGTATCAGGAACAGCTGGCCAATCTTGGCCCCGCCGGGCCAGACGGCAGTCTTC
CTCCGCTCCAGTACAAGGATCTTGACAAACTTCCCTTCCATCAACATGTTATTCGTGAAACCTTAC
GGATTCACTCCTCTATTCACTCTATCATGCGCAAGGTGAAAAGCCCCTTGCCCGTTCCCGGGAC
CCCTTACATGATTCCTCCCGGTCGCGTGCTCCTTGCTTCACCTGGAGTGACAGCCCTCAGCGA
CGAACACTTCCCCAATGCTGGGTGCTGGGATCCCCATCGCTGGGAGAACCAGGCTACTAAGG
AGCAGGAGAACGACGAGGTTGTCGACTACGGTTACGGCGCCGTCTCCAAGGGCACGTCAAGT
CCCTATCTTCCGTTTGGTGCTGGCCGACACCGCTGTATCGGCGAGAAATTCGCTTATGTCAACC
TTGGTGTGATTCTGGCGACCATTGTGCGCCACCTGCGACTTTTCAACGTGGATGGAAAGAAAGG
AGTCCCTGAAACTGACTATTCATCCCTCTTTTCGGGCCCCATGAAGCCAAGCATCATCGGCTGG
GAGAAGCGGTCGAAAAACACATCCAAGTGA

>MLI3796

ATTTACACTGGTTCTCCTCTAGAAAAAACTCATGAGTGAATAATCGCAGCACCACTTCAGAGTTGT
CTAGAATCACGCGGTCCGGATGTGTGCTGAGCCGAATGAAAGTTGCCTAATTACTAAGGTGAATA
ATTTACACTGCTCTCCTCTAAAAAAAACTCATGAGTGAATAATCTCATCACCACTTCACATTTGTCG
AGAATCACGCGAACCGGATGTGTGCTGAGCCGAATGAAATTTGCCTAATTACTAATGTGTAGTTCC
TGCATACCATACACCCTAACTCATACTACGGTAGGTACATCTACTTACCTATGAACCTATATCGGTA
GGTATGTGAATATAACAGACAGTATGGAACATGTTTTTCATTAGCTGGTCTCTCATTCGTCCTTGTCC
TAAGCCTTAAGGAATCCAGCATATGAAATAATCCCTCTTATACATTTTCCTCGTATTCTGTTTCATTTC
CCTCATCACTGCAACTCTAATCCTCGGGCTCACCCTCCCTGTGTCTCCTCTGAATGGTGCCGAT
GCTATGGCTTACGGCCTACATGGCCGTTGCGGTGCTGACGGCCATCTTGCTCAATGTTGTTTATC
AATTATTCTTTCGGCTTTGGAACCGAACAGAACCGCCAATGGTCTTTCATTGGGTCCCATTTCTGG
GTAGTACCATCAGTTACGGGATTGATCCCTACAAGTTCTTCTTTGCGTGCAGAGAAAAGGCAAGT
CTCAAGATTGTAGTTTGACATTCATTCCTGGGCGCATTGCTGAGTATTGCTTTCTTAACCGGCAGTA
TGGCGATATCTTCACTTTTATACTGTTGGGTCAAAAAACCACAGTCTACCTGGGCGTTCAGGGGA
ACGAGTTTATTCTCAACGGCAAGCTCAAGGATGTCAATGCGGAAGAGGTCTATAGTCCATTGACG
ACCCCCGTTTTCGGATCGGACGTGGTGTATGATTGTCCCAATTCCAAGCTGATGGAGCAGAAAA
AGTTCATCAAGTACGGCTTGACTCAGTCTGCGTTAGAGTCTCATGTGCCACTTATTGAGAAGGAG
GTTTTGGACTATCTGCGCGATTCACCGAACTTTCAAGGCTCGTCCGGCCGGATGGACATCTCTG
CGGCAATGGCTGAGATTACCATTTTTACCGCTGCTCGAGCCCTCCAAGGCCAGGAAGTTCGTTC
CAAACTCACGGCTGAGTTCGCTGACCTCTATCATGACCTGGACAAGGGCTTTACTCCCATCAATT
TTATGCTACCGTGGGCCCCATTGCCGCATAACAAGAAGCGAGATGCTGCTCATGCGCGCATGA
GGTCAATCTACGTTGACATCATCAATCAGCGCCGTCTTGACGGTGACAAGGACTCTCAGAAATCA
GACATGATATGGAACCTGATGAACTGCACATACAAAAACGGCCAGCAAGTGCCTGATAAAGAGA
TTGCGCACATGATGATAACCCTGTTGATGGCTGGTCAGCATTCGTCTTCGTCCATCAGCGCCTGG
ATTATGCTGAGACTGGCCTCACAGCCAAAAGTCCTCGAAGAGCTGTATCAGGAACAGCTGGCC
AATCTTGGCCCCGCCGGGCCAGACGGCAGTCTTCCTCCGCTCCAGTACAAGGATCTTGACAA
ACTTCCCTTCCATCAACATGTTATTCGTGAAACCTTACGGATTCACTCCTCTATTCACTCTATCATG
CGCAAGGTGAAAAGCCCCTTGCCCGTTCCCGGGACCCCTTACATGATTCCTCCCGGTCGCGT
GCTCCTTGCTTCACCTGGAGTGACAGCCCTCAGCGACGAACACTTCCCCAATGCTGGGTGCTG
GGATCCCCATCGCTGGGAGAACCAGGCTACTAAGGAGCAGGAGAACGACGAGGTTGTCGACT
ACGGTTACGGCGCCGTCTCCAAGGGCACGTCAAGTCCCTATCTTCCGTTTGGTGCTGGCCGAC
ACCGCTGTATCGGCGAGAAATTCGCTTATGTCAACCTTGGTGTGATTCTGGCGACCATTGTGCGC
CACCTGCGACTTTTCAACGTGGATGGAAAGAAAGGAGTCCCTGAAACTGACTATTCATCCCTCTT
TTCGGGCCCCATGAAGCCAAGCATCATCGGCTGGGAGAAGCGGTCGAAAAACACATCCAAGT
GAGACTGTTGTAACCATCGAGGACTTCA

>MLI3869

ATTTACACTGTTCTCCTCTAGAAAAACTCATGAGTGAATAATCGCAGCACCACTTCAGAGTTGTCTA
GAATCACGCGGTCCGGATGTGTGCTGAGCCGAATGAAAGTTGCCTAATTACTAAGGTGTAGTTCC
AGCATACCATACACCCTAACTCATACTACGGTAGGTAGATCTACTTACCTATGAACCTATATTGGTA
GGTAGGTGAATATAAAATACAGCATGGAACATGTTTTTCATTAGCTGGTCTCTCATTCGTCCTTGTC
CTAGGCCTTAAGGAATCCAGTATATGAAATAATCCCTCTTATCCATTTTCCTCCTATTCTTTTTCATTT
CCCTCATCACTGCAACTCTAATCCTCGGGCTCACCCTCCCTGTGTCTCCTCGAAATGGTGCCGA
TGCTATGGCTTACGGCCTACATGGCCGTTGCGGTGCTGACGGCAATCTTGCTCAATGTTGTTTAT
CAATTATTCTTTCGGCTTTGGAACCGAACAGAACCGCCAATGGTCTTTCATTGGGTCCCATTTCTG
GGTAGTACCATCAGTTACGGGATTGATCCCTACAAGTTCTTCTTTGCGTGCAGAGAAAAGGCAAG
TCTCAAGATTGTAGTTTGACATTCATTCCTGGGCGCATTGCTGAGTATTGCTTTCTTAACCGGCAGT
ATGGCGATATCTTCACTTTTATACTGTTGGGTCAAAAAACCACAGTCTACCTGGGCGTTCAGGGG
AACGAGTTTATTCTCAACGGCAAGCTCAAGGATGTCAATGCGGAAGAGGTCTATAGTCCATTGAC
GACCCCCGTTTTCGGATCGGACGTGGTGTATGATTGTCCCAATTCCAAGCTGATGGAGCAGAAA
AAGTTCATCAAGTACGGCTTGACTCAGTCTGCGTTAGAGTCTCATGTGCCACTTATTGAGAAGGAG
GTTTTGGACTATCTGCGCGATTCACCGAACTTTCAAGGCTCGTCCGGCCGGATGGACATCTCTG
CGGCAATGGCTGAGATTACCATTTTTACCGCTGCTCGAGCCCTCCAAGGCCAGGAAGTTCGTTC
CAAACTCACGGCTGAGTTCGCTGACCTCTATCATGACCTGGACAAGGGCTTTACTCCCATCAATT
TTATGCTACCGTGGGCCCCATTGCCGCATAACAAGAAGCGAGATGCTGCTCATGCGCGCATGA
GGTCAATCTACGTTGACATCATCAATCAGCGCCGTCTTGACGGTGACAAGGACTCTCAGAAATCA
GACATGATATGGAACCTGATGAACTGCACATACAAAAACGGCCAGCAAGTGCCTGATAAAGAGA
TTGCGCACATGATGATAACCCTGTTGATGGCTGGTCAGCATTCGTCTTCGTCCATCAGCGCCTGG
ATTATGCTGAGACTGGCCTCACAGCCAAAAGTCCTCGAAGAGCTGTATCAGGAACAGCTGGCC
AATCTTGGCCCCGCCGGGCCAGACGGCAGTCTTCCTCCGCTCCAGTACAAGGATCTTGACAA
ACTTCCCTTCCATCAACATGTTATTCGTGAAACCTTACGGATTCACTCCTCTATTCACTCTATCATG
CGCAAGGTGAAAAGCCCCTTGCCCGTTCCCGGGACCCCTTACATGATTCCTCCCGGTCGCGT
GCTCCTTGCTTCACCTGGAGTGACAGCCCTCAGCGACGAACACTTCCCCAATGCTGGGTGCTG
GGATCCCCATCGCTGGGAGAACCAGGCTACTAAGGAGCAGGAGAACGACGAGGTTGTCGACT
ACGGTTACGGCGCCGTCTCCAAGGGCACGTCAAGTCCCTATCTTCCGTTTGGTGCTGGCCGAC
ACCGCTGTATCGGCGAGAAATTCGCTTATGTCAACCTTGGTGTGATTCTGGCGACCATTGTGCGC
CACCTGCGACTTTTCAACGTGGATGGAAAGAAAGGAGTCCCTGAAACTGACTATTCATCCCTCTT
TTCGGGCCCCATGAAGCCAAGCATCATCGGCTGGGAGAAGCGGTCGAAAAACACATCCAAGT
GAGACTGTTGTAACCATCGAGGACTTCAAA
