# Supplementary material for: Azole Resistance in Aspergillus fumigatus From Diverse Environments in Ohio, United States, Is Primarily Driven by TR34/L98H and TR46/Y121F/T289A Environmental Signatures
Source: Open Forum Infect Dis. 2026 Apr 21;13(4):ofag150. doi: 10.1093/ofid/ofag150 (PMC13095377; doi:10.1093/ofid/ofag150)
Supplement: ofag150_Supplementary_Data [file ofag150_supplementary_data.zip › PAUL ET AL Supplementary Figure S1.pdf]

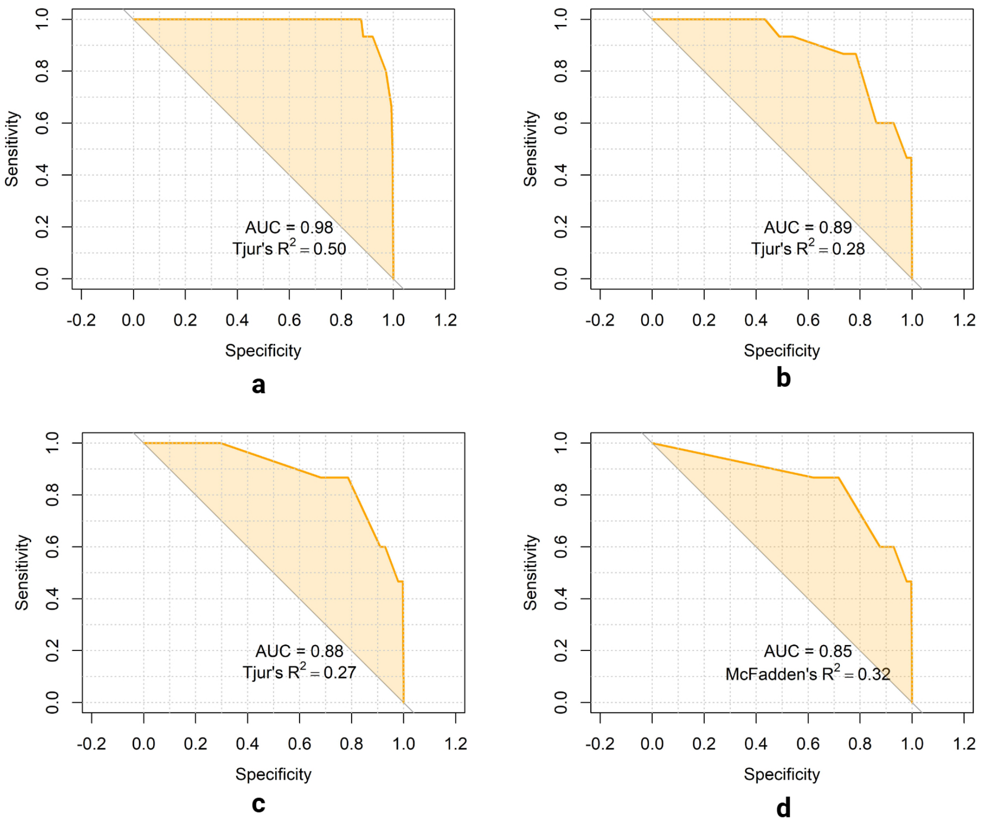

Supplementary Figure S1. Receiver-operating characteristic (ROC) curve analysis to assess the performance of predictive models for environmental ARAF. (a-c) generalized linear model (GLM), and d) GLM with LASSO regularization.
