# Supplementary material for: Azole Resistance in Aspergillus fumigatus From Diverse Environments in Ohio, United States, Is Primarily Driven by TR34/L98H and TR46/Y121F/T289A Environmental Signatures
Source: Open Forum Infect Dis. 2026 Apr 21;13(4):ofag150. doi: 10.1093/ofid/ofag150 (PMC13095377; doi:10.1093/ofid/ofag150)
Supplement: ofag150_Supplementary_Data [file ofag150_supplementary_data.zip › PAUL ET AL Supplementary Methods.pdf]

Azole-resistance in Aspergillus fumigatus from diverse environments in Ohio, United States is primarily driven by TR_{34}/L98H and TR_{46}/Y121F/T289A environmental signatures

Supplementary Methods

Sampling sites and sample collection

Agricultural environments included vineyards, vegetable farms, flower farms, apple orchards, corn and soybean fields, and small fruit farms. Golf courses, sport fields (excluding golf courses), city parks, and residential lawns were included in urban environments. Naturalized environments included woodlands/state parks, prairies, freshwater beaches, and wildlife areas; while the commercial environment sampled was an open-windrow composting facility. The air sampler was run for 10 minutes at three different locations at each site and soil (10 cm cores) from the same locations were also collected. At sites where in-house compost was present, 5-10 g of compost were collected from the compost pile. The efficiency of the air sampler to capture *A. fumigatus* spores and optimum operational time were first tested at an open windrow composting plant (CFAES-Wooster, Wooster, OH) using three different capture times (1 minute, 5 minutes, and 10 minutes) and five replicates for each time.

ARAF detection using culture

<u>Replica plating method optimization</u>

A. fumigatus conidia from reference isolates with WT *cyp*51A (AR0733, CDC and FDA Antimicrobial Resistance Bank), TR_{34}/L98H (AR0733, CDC and FDA Antimicrobial Resistance Bank), and TR_{46}/Y121F/T289A (AF-385, M.T. Brewer, University of Georgia, Athens, GA) were spiked in 1 mL sterile soil sample suspensions with a final conidial density as 1, 10, 100, 1000, and 10, 000 CFU/mL. A 200 µL aliquot from each spiked soil suspension was then plated onto control SDA and SDA amended with 5 µg/mL of propiconazole (SDAP) and incubated at 43°C for 48h. Reference isolates AR0733, and AF-385 exhibited growth on both SDAP and control SDA. This method proved to be sustainable, cost-effective, and less labor-intensive for the rapid screening of soil and spore suspensions with putative TR_{34}/L98H and TR_{46}/Y121F/T289A mutations. The lowest detection limit of this method was 10 CFU/mL for TR_{34}/L98H and TR_{46}/Y121F/T289A with 100% specificity (data not shown).

*cyp*51A tandem repeat (TR) detection from air and soil matrices

<u>DNA extraction</u>

Air samples suspended in PBS were centrifuged at 13800 × g for 5 minutes, and the pellet was resuspended in 0.8 ml of CD1 buffer. For the soil and compost samples (250 mg) were directly

dispended into the Power Bead Pro tubes and suspended in 0.8 ml of CD1 buffer. After brief vortexing, the suspensions were transferred to PowerBead Pro tubes and homogenized using a Powerlyzer™-24 (MO BIO Laboratories, Inc., Carlsbad, CA, USA) at a cycle speed of 3000 for 45 seconds followed by a 10-second pause, repeated thrice.

Nested PCR assay for TR mutation detection

In the first round of amplification, external primers, CypA-TR-S1 (5`-GGA GAA GGA AAG AAG CAC TCT-3`) and CypA-TR-AS1 (5'-TCA CCT ACC TAC CAA TAT AGG-3') were used, followed by a second round with internal primers, CypA-TR-S_A (5`-AGC ACCACT TCA GAG TTG TCT A-3`) and CypA-TR-AS_A (5`-TGT ATG GTA TGC TGGAAC TAC ACC TT-3`). The amplicons from the first round of PCR were diluted 1:20 in nuclease-free water and used as template for the second round of amplification. DNA from reference *A. fumigatus* isolates, AR0733, AR0740, and AF-385 served as controls for TR_{34}, TR_{46}, and WT *cyp*51A, respectively. The nested PCR products were separated by 2.5% agarose gel electrophoresis (80V for 2 hr.), stained with Gel Red Nucleic Acid Stain (Millipore Sigma, Merck KGaA, Darmstadt, Germany) and imaged using a Axygen Gel Documentation System (Corning Incorporated Life Sciences, Tewksbury, MA, USA).

Minimum Inhibitory Concentration Assays

All the triazole compounds were procured from Sigma Aldrich (St. Louis, MO, USA). A 100 µL aliquot of antifungal solution was pre-added to each well that would result in final concentration ranges of 0.13–64.00 µg/mL for the DMI fungicides and 0.06–32 µg/mL for the clinical azoles. Isolates were cultured on SDA at 37°C for 48 hr., after which conidia were harvested in 2 mL of 0.05% Tween-20 in PBS. The conidial suspensions were adjusted to an optical density of 0.09–0.13 at 530 nm, diluted 1:50 in RPMI-1640 liquid medium (Gibco, Thermo Fisher Scientific, Waltham, MA, USA) resulting in a final spore concentration of 10^4 CFU/mL. The MIC assay was performed in triplicate for all the isolates. Plates were incubated at 35°C for 48 hr. and MIC values, defined as the lowest azole concentration that completely inhibited the growth of *A. fumigatus*, were determined with optical density measurements at 530 nm.

Detection of *cyp51A* environmental signature mutations in ARAF isolates

DNA extraction from *A. fumigatus* isolates

Isolates were revived from silica bead stocks stored at −80°C on SDA supplemented with streptomycin sulfate (100 µg/mL) and chloramphenicol (50 µg/mL) in vented 25 cm^2 tissue culture flasks (Fisher Scientific, Pittsburgh, PA, USA) to promote sporulation and prevent contamination. After 48 hr. of incubation at 37°C, conidia were dislodged in 5 mL PBS containing 0.05% Tween-20 using a sterile loop and then filtered through a 40 µm cell strainer

(Thomas Scientific, Irvine, CA, USA) to remove any mycelial fragments. A 1.5 mL aliquot of the filtered conidial suspension was centrifuged at 7200 × g for 10 minutes. The pellet was resuspended in 300 µL of Tissue and Cell Lysis Solution and transferred to 2 mL FastPrep tubes with 1 mm silica beads (MP Biomedicals, Irvine, CA, USA). Homogenization was performed using the PowerLyzer-24, as described above. The lysate was centrifuged at 16200 × g, and the supernatant was treated with RNase A at 65°C for 15 minutes followed by cooling on ice. DNA purification continued using the DNeasy Blood and Tissue kit (Qiagen, Germantown, MD, USA)

*cyp51*A gene sequencing

*cyp5*1A gene was amplified in a 25 µL PCR reaction Hot Start High-Fidelity Mastermix (New England BioLabs, Ipswich, MA, USA) and 0.25 µM of each primer. The thermal cycling was performed with an initial denaturation at 98°C, followed by 30 cycles of 98°C for 30 seconds, 65°C for 15 seconds, and 72°C for 2 minutes. A 5 µL aliquot of the PCR product was purified using 2 µL of ExoSAP II PCR product clean-up reagent (Applied Biosystems, Waltham, MA, USA) following the manufacturer's instructions. The purified products were then diluted to 20 µL and sequenced at Eurofins Genomics LLC (Louisville, KY, USA).
