# Supplementary material for: Azole Resistance in Aspergillus fumigatus From Diverse Environments in Ohio, United States, Is Primarily Driven by TR34/L98H and TR46/Y121F/T289A Environmental Signatures
Source: Open Forum Infect Dis. 2026 Apr 21;13(4):ofag150. doi: 10.1093/ofid/ofag150 (PMC13095377; doi:10.1093/ofid/ofag150)
Supplement: ofag150_Supplementary_Data [file ofag150_supplementary_data.zip › PAUL ET AL Supplementary Table S1.pdf]

Azole-resistance in Aspergillus fumigatus from diverse environments in Ohio, United States is primarily driven by TR_{34}/L98H and TR_{46}/Y121F/T289A environmental signatures

Supplementary Table S1. Classification of sampling environments, sample types, and number of samples collected

Environment category	Environment subcategory	N of sampling sites	N of airborne spore samples	N of soil/compost samples
Agricultural	Vineyards	10	50	50
	Vegetable farms	12	57	58
	Cut flower farms	8	43	45
	Apple orchards	9	54	53
	Corn and soybean farms	3	30	30
	Small fruit farms	2	10	10
	**Total**	**44**	**244**	**246**
Urban	Golf courses	5	27	41
	Sport fields (excluding golf courses)	3	15	15
	City parks	1	6	5
	Residential lawns	2	10	12
	**Total**	**11**	**58**	**73**
Naturalized	Woodlands/State parks	13	65	40
	Prairies	3	15	15
	Freshwater beaches	2	10	10
	Wetlands	1	5	5
	**Total**	**19**	**95**	**75**
Commercial	Open windrow composting facility	**1**	**14**[*]	**0**

[*]These samples were used in the optimization of the air sampling (spore capture) procedure; nested-PCR was not conducted on these samples.
